# Identification of Seven Key Structural Genes in the Anthocyanin Biosynthesis Pathway in Sepals of *Hydrangea macrophylla*

**DOI:** 10.3390/cimb44090286

**Published:** 2022-09-12

**Authors:** Hui Qi, Gaitian Zhang, Zhiyun Chu, Chun Liu, Suxia Yuan

**Affiliations:** Institute of Vegetables and Flowers, Chinese Academy of Agricultural Sciences, Beijing 100081, China

**Keywords:** full-length transcriptome, total anthocyanin Delphinidin 3-glucoside, gene expression

## Abstract

Under specific cultivation conditions, the sepal color of *Hydrangea macrophylla* (*H.* *macrophylla*) changes from red to blue due to the complexation of aluminum ions (Al^3+^), delphinidin 3-glucoside, and copigments. However, this phenomenon cannot occur in all cultivars despite the presence of sufficient Al^3+^ and copigments. To explore the mechanism of sepal bluing in *H. macrophylla*, there is an urgent need to study the molecular regulation of the anthocyanin biosynthesis pathway. However, the key structural genes, other than *CHS*, regulating anthocyanin biosynthesis in the sepals of *H. macrophylla* have not been identified. In this study, based on full-length transcriptome data from *H.* *macrophylla* ‘Bailmer’, the key structural genes regulating anthocyanin biosynthesis in the sepals of *H. macrophylla* were isolated and investigated. Ultimately, seven key structural genes, *HmCHS1*, *HmCHI*, *HmF3H1*, *HmF3*′*H1*, *HmF3*′*5*′*H*, *HmDFR2*, and *HmANS3*, were demonstrated to show high expression levels in colored sepals. The expression levels of these seven genes increased gradually with the development of sepals and were highest in the full-bloom stage. The trend of gene expression was consistent with the trend of anthocyanin contents. It was concluded that the seven selected genes were involved in anthocyanin biosynthesis in the sepals of *H. macrophylla*. The full-length sequence data have been deposited into the NCBI Sequence Read Archive (SRA) with accession number PRJNA849710. This study lays a good foundation for the further elucidation of the molecular mechanism of sepal coloration in *H.* *macrophylla*.

## 1. Introduction

*Hydrangea macrophylla* (*H. macrophylla*) is a deciduous shrub in the family Hydrangeaceae. *H. macrophylla* is a popular ornamental plant due to its large inflorescences, various flower colors, and long flowering period; therefore, it is widely used for cut flower production and as a potted or landscape plant. The sepals of *H. macrophylla* are its main ornamental organs [1]. The colors of *H. macrophylla* sepals vary from white to pink, red, purple, and blue, among which blue sepals are popular with consumers in the market. Interestingly, the development of blue sepals depends on cultivation conditions. In suitable acidic soil, aluminum ions (Al^3+^) can be taken up by the roots and then transported into the vacuoles of sepals, where they form complexes with anthocyanins and copigments; thus, the sepal color changes from the original red coloration to purple and then blue [2,3,4,5]. In alkaline soil, aluminum exists as an Al(OH)_3_ precipitate and cannot be taken up, so the sepals retain their original color of red or pink [6,7,8].

Because of this unique characteristic, the mechanism of sepal color formation has become a research hotspot. Initially, it was found that the anthocyanin 3-*O*-glucosyldelphinidin and the copigment 5-*O*-caffeoylquinic acid or 5-*O*-p-coumaroylquinic acid were involved in the development of blue sepals [9,10,11,12,13]. Then, it was reported that the vacuole pH value also played a key role in coloration, as the pH levels of the blue and red vacuoles were significantly different, at 4.1 and 3.3, respectively. When 3-*O*-glucosyldelphinidin, 5-*O*-caffeoylquinic acid or 5-*O*-p-coumaroylquinic acid and Al^3+^ were mixed in buffer solution at a ratio of 1:1:1 at pH 4.0, the blue color could be reproduced in vitro [14], revealing the molecular structure of the blue metal complex pigment in *H. macrophylla*. A recent study indicated that the blue cells in the sepals were mainly located in the second cell layer and that the distributions of Al^3+^ and the *H. macrophylla* blue complex overlapped with the area of blue cells, which confirmed that aluminum and the *H. macrophylla* blue-complex exist in the blue cells of sepals and are involved in blue coloration [15].

Although the molecules included in the *H. macrophylla* blue complex have been identified, it is still difficult to regulate blue flower production, and not all cultivars can be induced to develop a blue color under specific cultivation conditions. At present, the molecular mechanism of flower color formation in sepals is still unclear.

In general, red and blue colors are determined by pelargonidins and delphinidins, respectively [10,16]. However, in *H. macrophylla*, all sepal colors originate from a common anthocyanin, 3-*O*-glucosyldelphinidin [9,14]. The anthocyanin biosynthesis pathway has been well studied as an important pathway of a branch of the plant flavonoid pathway; there are seven key structural genes in the anthocyanin biosynthesis pathway, including the chalcone synthase (*CHS*), chalcone isomerase (*CHI*), flavonoid 3-hydroxylase (*F3H*), flavonoid 3-hydroxylase (*F3*′*H*), flavonoid 3-5 hydroxylase (*F3*′*5*′*H*), dihydroflavonol 4-reductase (*DFR*), and anthocyanidin synthase (*ANS*) genes [17,18]. Although *CHS*, *DFR*, and *F3H* have been reported to regulate anthocyanin biosynthesis in the sepals of *H. macrophylla*, sequence information was found only for *CHS* [19,20,21]. To gain further insight into the molecular mechanism of sepal coloration, it is essential to determine the key structural genes regulating anthocyanin biosynthesis in the sepals of *H. macrophylla*. In this study, based on full-length transcriptome data from *H. macrophylla* ‘Bailmer’, the *CHS*, *CHI*, *F3H*, *F3*′*H*, *F3*′*5*′*H*, *DFR*, and *ANS* genes were selected as candidate genes, and the key structural genes regulating anthocyanin biosynthesis in sepals were identified.

## 2. Materials and Methods

### 2.1. Plant Materials and Sampling

Three cultivars of *H. macrophylla* were used in this research, among which ‘Bailmer’ and ‘Duro’ are cultivars with pink sepals, whereas ‘Saxon Kleiner Winterberg’ has white sepals. Under aluminum sulfate treatment, the sepal color of ‘Bailmer’ turned blue, while that of ‘Duro’ and ‘Saxon Kleiner Winterberg’ remained pink and white, respectively. These cultivars were planted in the greenhouse at the Institute of Vegetables and Flowers, Chinese Academy of Agricultural Sciences (Beijing, China). Half of the ‘Bailmer’ plants were treated with an aluminum sulfate solution (Al_2_(SO4)_3_·18H_2_O dissolved in water), and the sepals turned blue (Tr group). The substrate mixture was soaked with the aluminum sulfate solution 9 times, and each pot was soaked in approximately 20 g of aluminum sulfate (Al_2_(SO4)_3_·18H_2_O) in total. The remaining half of the ‘Bailmer’ plants (CK group) and all ‘Duro’ and ‘Saxon Kleiner Winterberg’ plants were grown without aluminum sulfate treatment.

**Figure 1:**
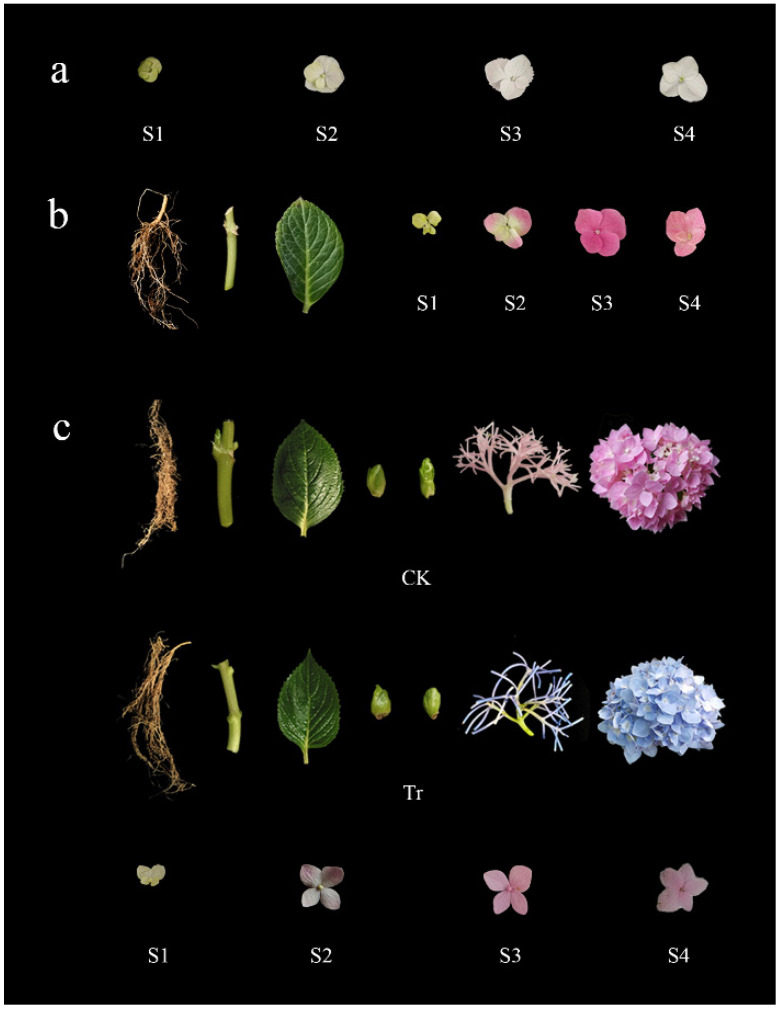
Materials of ‘Saxon Kleiner Winterberg’, ‘Duro’, and ‘Bailmer’. (**a**) Sepals in four developmental stages (S1, S2, S3, and S4) from ‘Saxon Kleiner Winterberg’; (**b**) roots, stem, leaf, and sepals in four developmental stages (S1, S2, S3, and S4) from ‘Duro’; (**c**) roots, stems, leaves, terminal buds, lateral buds, pedicels, and flowers from the CK and Tr groups of ‘Bailmer’ and sepals in four developmental stages (S1, S2, S3, and S4) from the CK group of ‘Bailmer’.

The roots, stems, leaves, terminal buds, lateral buds, pedicels, flowers, and sepals were harvested at four developmental stages (S1: bud stage sepals, S2: discoloration stage sepals, S3: full-bloom stage sepals, S4: senescence stage sepals) (Figure 1). All of these plant materials were immediately frozen in liquid nitrogen and then stored at −80 °C.

### 2.2. Determination of Total Anthocyanin Contents

For ‘Saxon Kleiner Winterberg’, ‘Duro’, and ‘Bailmer’ (CK), the total anthocyanin contents of the sepals in four developmental stages (S1, S2, S3, and S4) were determined using a Plant Polyphenol-Chlorophyll Measuring Instrument (Dualex Scientific+, Orsay, France).

### 2.3. Full-Length Transcriptome Sequencing and Gene Annotation

Total RNA was extracted from the roots, stems, leaves, terminal buds, lateral buds, pedicels, and flowers of the CK group and Tr group of ‘Bailmer’ by grinding the tissue in TRIzol reagent (Life Technologies, Carlsbad, CA, USA) on dry ice and processing it according to the protocol provided by the manufacturer. An Agilent^®^ 2100 Bioanalyzer (Agilent, Palo Alto, CA, USA) and a NanoDrop^®^ series spectrophotometer (Thermo Scientific, Carlsbad, CA, USA) were used to determine the integrity and concentration of the RNA samples. The qualified RNA samples were mixed in equal amounts. mRNA was then enriched by using Oligo (dT) magnetic beads, and the enriched 5 μg mRNA was reverse transcribed into first-strand cDNA using the Clontech SMARTer PCR cDNA Synthesis Kit (Clontech, San Francisco, CA, USA). Then, PCR amplification was performed to synthesize double-stranded cDNA, and AMPure PB Beads (AMPure, Pasadena, CA, USA) were used to purify PCR amplification products. The SMRTbell template was annealed to a sequencing primer and bound to polymerase, and sequencing was conducted on the PacBio SequelII platform by Gene Denovo Biotechnology Co. (Guangzhou, China). The raw sequencing reads of the cDNA libraries were analyzed using an isoform sequencing (Iso-Seq) pipeline supported by Pacific Biosciences [22].

To annotate isoforms, they were subjected to BLAST searches against the NCBI nonredundant protein (Nr) database (http://www.ncbi.nlm.nih.gov, accessed on 2 December 2020), the Swiss-Prot protein database (http://www.expasy.ch/sprot, accessed on 4 December 2020), the Kyoto Encyclopedia of Genes and Genomes (KEGG) database (http://www.genome.jp/kegg, accessed on 7 December 2020), and the COG/KOG database (http://www.ncbi.nlm.nih.gov/COG, accessed on 9 December 2020). The BLASTx program (http://www.ncbi.nlm.nih.gov/BLAST/, accessed on 26 December 2020) was run with an E-value threshold of 1 × 10^−5^ to evaluate sequence similarity with genes of other species.

### 2.4. Selection and Validation of Genes

#### 2.4.1. Gene Selection

*CHS*, *CHI*, *F3H*, *F3*′*H*, *F3*′*5*′*H*, *DFR*, and *ANS*, which encode major enzymes in the anthocyanin biosynthesis pathway, were searched based on full-length transcriptome data. The open reading frames (ORFs) of the isoforms were analyzed online with NCBI ORF finder software (https://www.ncbi.nlm.nih.gov/orffinder accessed on 1 September 2022, NCBI, Bethesda, USA), and the multiple sequence alignment of isoforms was carried out using DNAMAN software.

#### 2.4.2. qRT–PCR analysis

The roots, leaves, and sepals at S1, S2, and S3 in ‘Bailmer’, the roots, leaves, and sepals at S3 in ‘Duro’, and sepals at S3 in ‘Saxon Kleiner Winterberg’ from the CK group (without aluminum sulfate treatment) were harvested and were stored at −80 °C. All samples were ground to a powder in liquid nitrogen to extract total RNA using an RNA extraction kit (Huayueyang Biotechnology Inc., Beijing, China) according to the manufacturer’s instructions. The integrity and concentration of the RNA were analyzed by using 1% agarose gel electrophoresis and an Ultramicro ultraviolet-visible spectrophotometer (ND-100C, Miulab, Hangzhou, China), respectively. First-strand cDNA was synthesized using a PrimeScriptTM RT reagent kit with gDNA Eraser (Takara, Osaka, Japan) according to the manufacturer’s instructions.

The primer pairs targeting the genes were designed using Primer Premier 5.0 software (Premier, Ottawa, Canada) (Table 1). The *EF1-β* housekeeping gene (F- CGCAGCTGTTTTAGGGAAGCC, R- GCGAGCTGCGAAGACACAGA) was used as the internal control for normalization. The cDNA template was fully mixed with Taq Pro Universal SYBR qPCR Master Mix (Vazyme, Nanjing, China) for qRT–PCR in a Light Cycler 480 System (Roche, USA). The qRT–PCR protocol was as follows: 95 °C for 30 s, followed by 40 cycles of 95 °C for 10 s, and 60 °C for 30 s. Finally, melting curves were generated for all genes at 95 °C for 15 s, 60 °C for 60 s, and 95 °C for 15 s. The relative expression levels of the target genes were calculated via the 2^−ΔΔCq^ method. One-way ANOVA (significant differences *p* = 0.05), Duncan’s method for multiple comparison tests, and Pearson correlation analysis were performed using SPSS 2.0 software (IBM, Armonk, USA).

## 3. Results

### 3.1. Anthocyanin Contents

**Figure 2:**
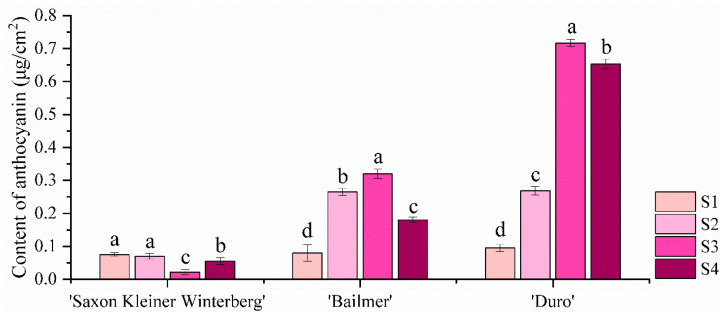
Contents of total anthocyanins in sepals at different developmental stages in ‘Saxon Kleiner Winterberg’, ‘Bailmer’(CK), and ‘Duro’. Different lowercase letters indicating significant differences at the 0.05 level of probability according to Duncan’s multiple-range test.

In the two cultivars with colored sepals (‘Bailmer’ and ‘Duro’), the contents of total anthocyanins in the sepals increased from the bud stage (S1) to the full-bloom stage (S3) and then decreased in the senescence stage (S4), with the highest accumulation of anthocyanins being observed at S3 (Figure 2). In ‘Saxon Kleiner Winterberg’ with white sepals, the anthocyanin content of the sepals remained low throughout their development.

### 3.2. Analysis of Full-Length Transcriptome Sequencing Data

**Figure 3:**
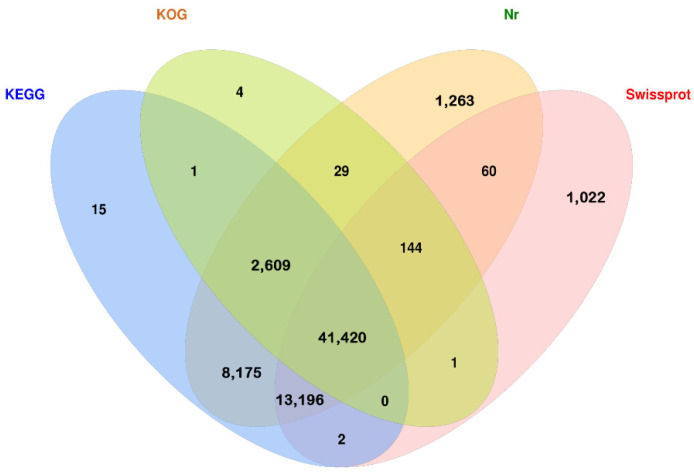
Venn diagram of isoform annotations according to four databases.

A total of 72,848 high-quality isoforms were obtained from the full-length transcriptome sequencing data. A total of 67,941 (93.26%) of these isoforms were annotated in the Nr, SwissProt, KEGG, and KOG databases. The specific numbers of isoforms annotated in the NR, KEGG, KOG and SwissProt databases were 66,896 (91.83%), 65,418 (89.80%), 44,208 (60.69%), and 55,845 (76.66%), respectively (Figure 3).

### 3.3. Screening of Key Structural Genes Involved in Anthocyanin Biosynthesis

Thirty-seven isoforms related to structural genes in the anthocyanin biosynthesis pathway that showed similar sequences to *CHS*, *CHI, F3H, F3*′*H, F3*′*5*′*H, DFR*, and *ANS* were identified via NCBI BLAST searches. The sequences of isoforms showing more than 98% coverage and 98% identities in the CDS region were classified as belonging to the same gene. Thus, a total of 14 genes were obtained (Table 1). All of the structural genes except for *CHI* and *F3*′*5*′*H* had multiple family members. The specific primer pairs used for each gene are listed in Table 1.

### 3.4. Expression Pattern Analysis of Key Structural Genes Involved in Anthocyanin Biosynthesis

**Figure 4:**
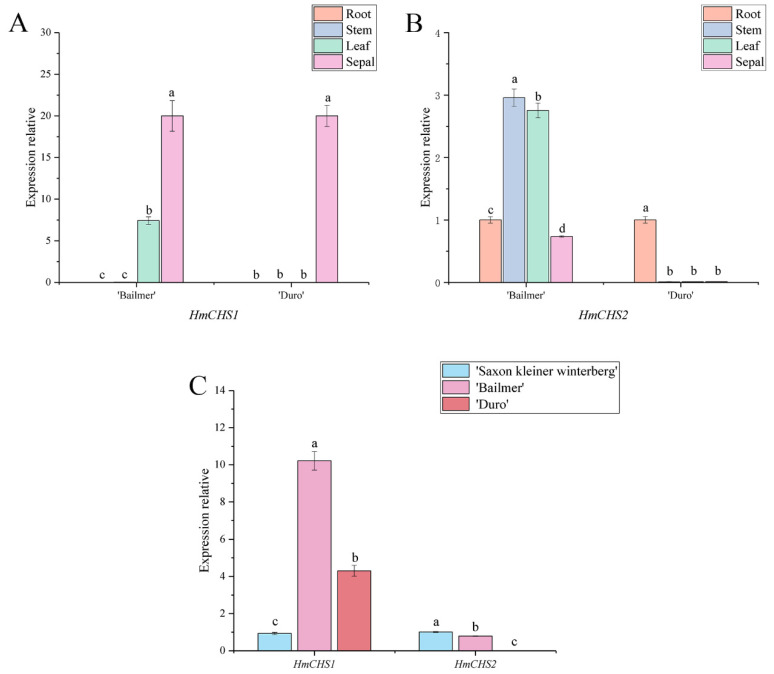
(**A**) Expression levels of *HmCHS1* in different organs; (**B**) expression levels of *HmCHS2* in different organs; (**C**) expression levels of *HmCHS1* and *HmCHS2* in sepals among the three cultivars. Different lowercase letters indicating significant differences at the 0.05 level of probability according to Duncan’s multiple-range test.

The *H. macrophylla CHS* gene family has two members, *HmCHS1* and *HmCHS2*. The expression patterns of *HmCHS1* in the roots, stems, leaves, and full-bloom stage sepals were different from those of *HmCHS2*. In both ‘Bailmer’ and ‘Duro’, the expression levels of *HmCHS1* were the highest in the sepals and lowest in the roots and stems (Figure 4A), whereas the expression level of *HmCHS2* in sepals was lower than that in the other organs (Figure 4B).

Subsequently, the expression levels of *HmCHS1* in the pink sepals of ‘Bailmer’ and ‘Duro’ were significantly higher than those in the white sepals of ‘Saxon Kleiner Winterberg’. However, the expression levels of *HmCHS2* in the sepals of ‘Bailmer’ and ‘Duro’ were lower than the level in ‘Saxon Kleiner Winterberg’ (Figure 4C).

The results indicated that *HmCHS1* is a key structural gene involved in anthocyanin biosynthesis in sepals.

**Figure 5:**
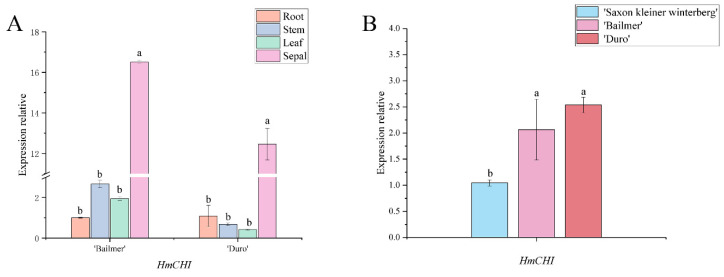
(**A**) Expression levels of *HmCHI* in different organs; (**B**) expression levels of *HmCHI* in sepals among the three cultivars. Different lowercase letters indicating significant differences at the 0.05 level of probability according to Duncan’s multiple-range test.

Only one *CHI* gene, named *HmCHI*, was obtained. In both ‘Bailmer’ and ‘Duro’, the expression levels of *HmCHI* were the highest in sepals among all the tested organs (Figure 5A). Moreover, the expression levels of *HmCHI* in the pink sepals of ‘Bailmer’ and ‘Duro’ were higher than those in the white sepals of ‘Saxon Kleiner Winterberg’ (Figure 5B). Therefore, *HmCHI* is the key structural gene involved in anthocyanin biosynthesis in sepals.

**Figure 6:**
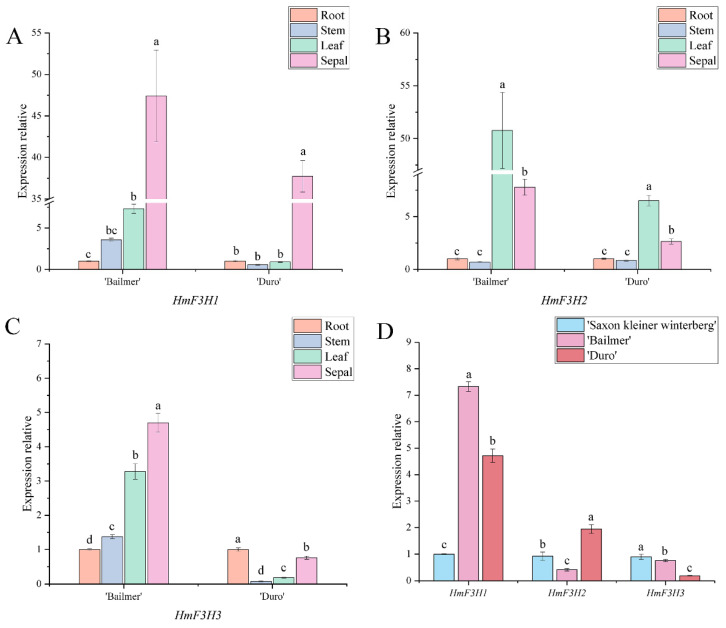
(**A**) Expression levels of *HmF3H1* in different organs; (**B**) expression levels of *HmF3H2* in different organs; (**C**) expression levels of *HmF3H3* in different organs; (**D**) expression levels of *HmF3H1*, *HmF3H2*, and *HmF3H3* in sepals among the three cultivars. Different lowercase letters indicating significant differences at the 0.05 level of probability according to Duncan’s multiple-range test.

There are three members of the *H. macrophylla F3H* gene family, *HmF3H1*, *HmF3H2*, and *HmF3H3*. In both ‘Bailmer’ and ‘Duro’, *HmF3H1* presented the highest expression level in sepals (Figure 6A), while *HmF3H2* showed the highest expression level in leaves. (Figure 6B). Although *HmF3H3* exhibited the highest expression level in the sepals of ‘Bailmer’, it exhibited the highest expression level in the roots of ‘Duro’ (Figure 6C). In addition, the expression levels of *HmF3H1* were significantly higher in pink sepals than in white sepals (Figure 6D). Therefore, *HmF3H1* is a key structural gene involved in anthocyanin biosynthesis in sepals.

**Figure 7:**
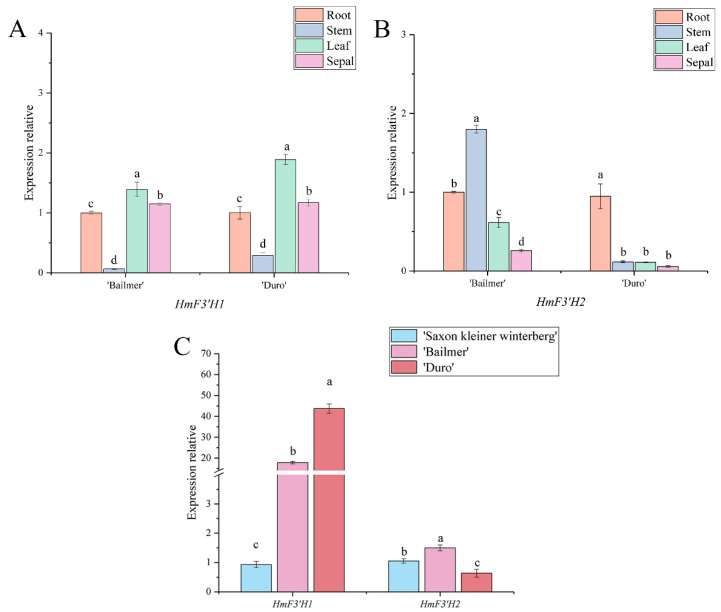
(**A**) Expression levels of *HmF3*′*H1* in different organs; (**B**) expression levels of *HmF3*′*H2* in different organs; (**C**) expression levels of *HmF3*′*H1* and *HmF3*′*H2* in sepals among the three cultivars. Different lowercase letters indicating significant differences at the 0.05 level of probability according to Duncan’s multiple-range test.

Two members of the *F3*′*H* gene family were identified, *HmF3*′*H1* and *HmF3*′*H2*. In ‘Bailmer’, the expression level of *HmF3*′*H1* was lower in sepals than in roots and leaves, and in ‘Duro’, its expression was lower in sepals than in leaves (Figure 7A). *HmF3*′*H2* presented the lowest expression level in sepals among all the tested organs (Figure 7B). Thus, the expression levels of *HmF3*′*H1* and *HmF3*′*H2* were not higher in sepals than in the other organs.

Furthermore, the expression levels of *HmF3*′*H1* in the colored sepals of the two cultivars were significantly higher than those in the white sepals of ‘Saxon Kleiner Winterberg’. However, the expression level of *HmF3*′*H2* in the pink sepals of ‘Duro’ was lower than that in the white sepals of ‘Saxon Kleiner Winterberg’ (Figure 7C).

In conclusion, *HmF3*′*H1* is a key structural gene involved in anthocyanin biosynthesis in sepals.

**Figure 8:**
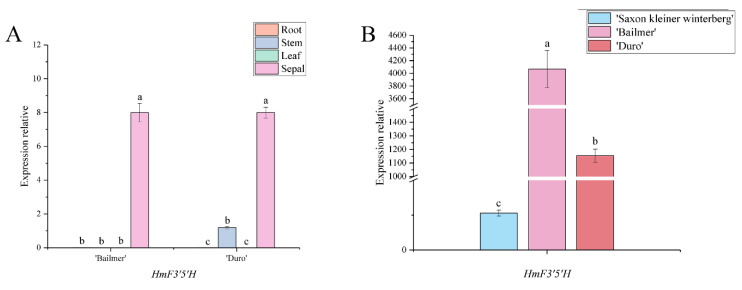
(**A**) Expression levels of *HmF3*′*5*′*H* in different organs; (**B**) expression levels of *HmF3*′*5*′*H* in sepals among the three cultivars. Different lowercase letters indicating significant differences at the 0.05 level of probability according to Duncan’s multiple-range test.

Only one gene annotated as *F3*′*5*′*H*, named *HmF3*′*5*′*H*, was screened. The expression level of *HmF3*′*5*′*H* was the highest in sepals among all the tested organs (Figure 8A). The expression level of *HmF3*′*5*′*H* was significantly higher in colored sepals than in white sepals (Figure 8B). Therefore, *HmF3*′*5*′*H* is a key structural gene involved in anthocyanin biosynthesis in sepals.

**Figure 9:**
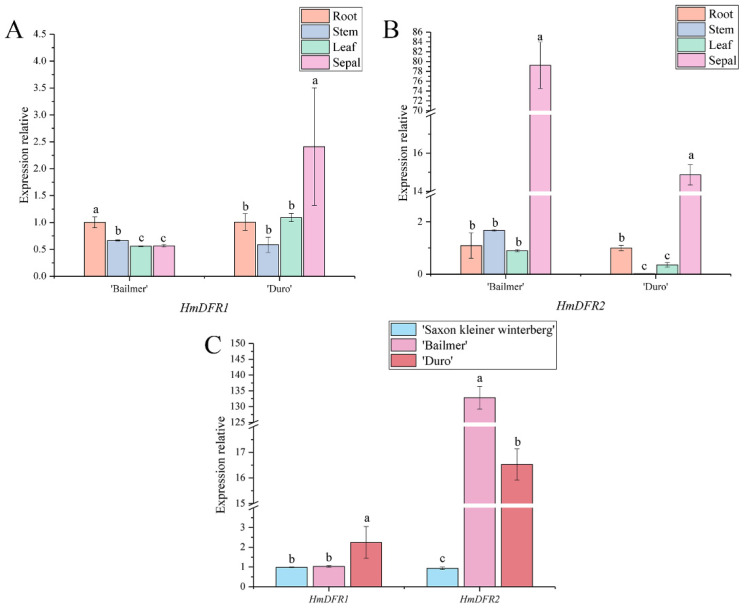
(**A**) Expression levels of *HmDFR1* in different organs; (**B**) expression levels of *HmDFR2* in different organs; (**C**) expression levels of *HmDFR1* and *HmDFR2* in sepals among the three cultivars. Different lowercase letters indicating significant differences at the 0.05 level of probability according to Duncan’s multiple-range test.

Two *DFR* genes, *HmDFR1* and *HmDFR2*, were found. The expression level of *HmDFR1* was highest in sepals in ‘Bailmer’, whereas its expression was lower in sepals than in roots and stems in ‘Duro’ (Figure 9A). However, the expression levels of *HmDFR2* were highest in sepals among all the examined organs of both ‘Bailmer’ and ‘Duro’ (Figure 9B).

Additionally, the expression level of *HmDFR2* in colored sepals was higher than that in white sepals (Figure 9C). However, there was no significant difference in the expression level of *HmDFR1* between colored sepals and white sepals.

It was concluded that *HmDFR2* is a key structural gene involved in anthocyanin biosynthesis in sepals.

**Figure 10:**
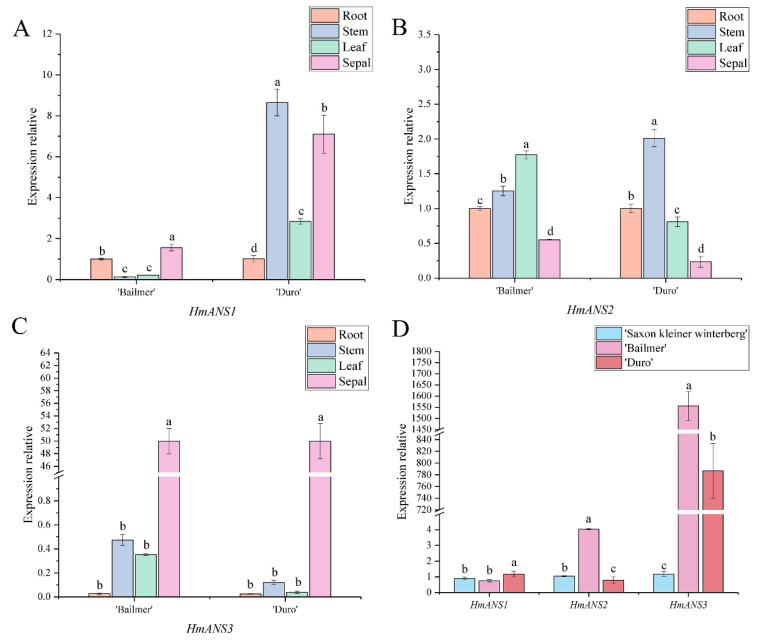
(**A**) Expression levels of *HmANS1* in different organs; (**B**) expression levels of *HmANS2* in different organs; (**C**) expression levels of *HmANS3* in different organs; (**D**) expression levels of *HmANS1, HmANS2*, and *HmANS3* in sepals among the three cultivars. Different lowercase letters indicating significant differences at the 0.05 level of probability according to Duncan’s multiple-range test. In summary, according to the expression patterns observed in different organs, and the differences in expression levels between colored sepals and white sepals, seven genes, *HmCHS1*, *HmCHI*, *HmF3H1*, *HmF3*′*H1*, *HmF3*′*5*′*H*, *HmDFR2*, and *HmANS3*, were identified as key structural genes involved in anthocyanin biosynthesis in sepals.

Similar to *F3H*, there were three identified members of the *ANS* gene family, *HmANS1*, *HmANS2*, and *HmANS3*. In ‘Bailmer’ and ‘Duro’, only *HmANS3* showed a higher expression level in sepals than in the other organs (Figure 10A–C). Moreover, the expression level of *HmANS3* was significantly higher in colored sepals than in white sepals (Figure 10D).

Therefore, *HmANS3* is a key structural gene involved in anthocyanin biosynthesis in sepals.

### 3.5. Expression Patterns of the Seven Structural Genes Involved in Anthocyanin Biosynthesis during the Development of Sepals

**Figure 11:**
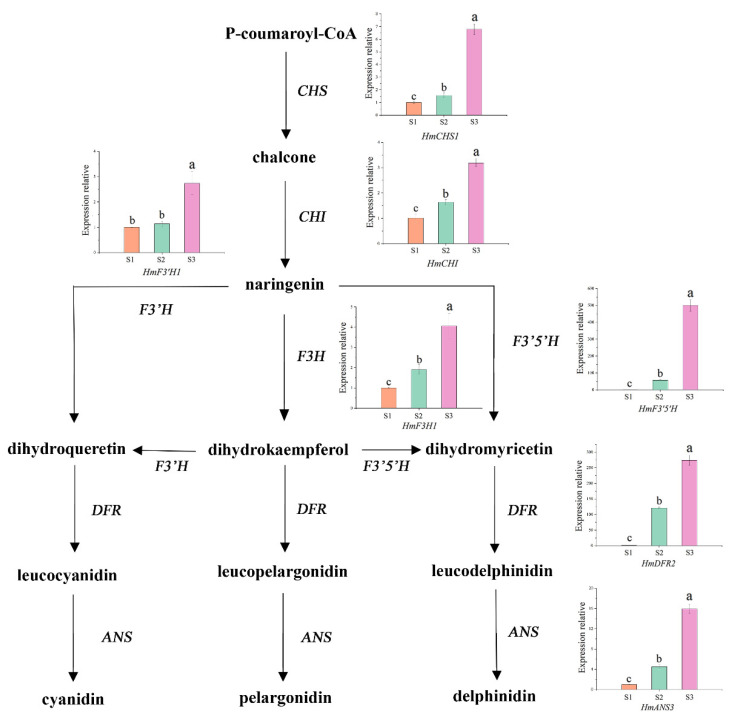
Expression patterns of seven structural genes in the three developmental stages (S1, S2, S3) of sepals. Different lowercase letters indicating significant differences at the 0.05 level of probability according to Duncan’s multiple-range test.

In ‘Bailmer’ (CK) sepals, the expression levels of *HmCHS1*, *HmCHI*, *HmF3H1*, *HmF3*′*H1*, *HmF3*′*5*′*H*, *HmDFR2*, and *HmANS3* increased gradually with the development of sepals (from S1 to S2, S3) and peaked at the full-bloom stage (S3) (Figure 11). The trend of gene expression was consistent with the trend of the anthocyanin contents (*p* < 0.01; Table 2). It was proven that the seven selected genes were related to anthocyanin biosynthesis in sepals.

## 4. Discussion

Anthocyanin accumulates in the vacuoles of petals and sepals and is the main pigment involved in flower color formation [7,23,24]. In the *H. macrophylla* ‘Meihong Mother’ cultivar, the anthocyanin content of sepals increases from the bud stage to the full-bloom stage and then decreases in the senescence stage. The accumulation of anthocyanin has also been reported to be highest in the full-flower stage in the ‘Thunb Ser’, ‘EndLess Summer’, and ‘Emile Mouillere’ cultivars [20,25]. In this study, the contents of the total anthocyanins in the colored sepals of ‘Bailmer’ and ‘Duro’ were similarly found to increase gradually from S1 to S2 and S3 and then decrease in S4 (Figure 2).

Although the genomics of *H. macrophylla* are being studied, and many complete or partial nucleotide sequences have been published in public databases [26], transcriptome sequencing remains an efficient method for mining genes. In our study, ‘Bailmer’ was selected for full-length transcriptome sequencing because of its unique characteristics, including blooming in new growth without a cold requirement, low-temperature resistance, and the development of blue flowers in acidic soil containing Al^3+^. The full-length transcriptome database is helpful for exploring the mechanisms of flower color formation and aluminum detoxification in *H. macrophylla*. In the full-length transcriptome database, the *CHS*, *F3H*, *F3*′*H*, *DFR*, and *ANS* gene families showed multiple members, whereas *CHI* and *F3*′*5*′*H* did not (Table 1). Similar to many other higher plants, genome evolution has produced gene families by gene duplication in this species [27]. In *Chrysanthemum*, there are multiple members of the structural gene families involved in the flavonoid biosynthesis pathway, with the exception of *F3*′*5*′*H* and *ANS* [28]. In *lily*, four unigenes have been annotated as *CHS* genes [29]. In the *H. macrophylla* ‘Forever Summer’ cultivar, multiple members have been identified for all key structural gene families except for *CHI* [30]. In this study, *HmCHS1*, *HmCHI*, *HmF3H1*, *HmF3*′*5*′*H*, *HmDFR2*, and *HmANS3* were selected as key structural genes participating in anthocyanin biosynthesis in sepals, and other members of their gene families may participate in flavonoid biosynthesis pathways in other tissues of *H. macrophylla*.

*CHS* [19], *DFR* [20], and *F3H* [21] have been reported to be structural genes related to anthocyanin biosynthesis in *H. macrophylla*. The sequence of *HmCHS*1 identified in this experiment was highly similar (98.82%) to that of the *CHS* gene (AB011467.1) based on sequence comparison; thus, they should be identified as the same gene. However, there is a lack of sequence information about *F3H* and *DFR* in the literature, and we cannot determine whether the sequences of *HmF3H1* and *HmDFR2* selected in this study are the same as genes in previous reports.

Anthocyanins are the main compounds responsible for flower coloration, so the expression levels of structural genes regulating anthocyanin biosynthesis in sepals should be higher than those in other organs. All of the structural genes identified in this work except for *HmF3*′*H1* showed the highest expression levels in sepals. The two members of the F3′H family, both *HmF3*′*H1* and *HmF3*′*H2*, did not show higher expression in sepals than in the other organs. A similar result was found in *Camellia Nitidissima Chi*, in which the expression level of the *F3*′*H* gene in petals was lower than that in leaves, whereas it was highest in fruits [31]. In *H. macrophylla*, *HmF3*′*H1* and *HmF3*′*H2* may play vital roles in other biosynthesis processes in leaves, stems, and roots.

## Figures and Tables

**Table 1:** The structural genes in the anthocyanin biosynthesis pathway in *H. macrophylla* and the corresponding primer sequences.

Protein Name	Gene Name	Family Member	Isoforms ID	Primer Sequence (5′-3′)	Amplicon Length
Chalcone synthase	*CHS*	*HmCHS1*	Isoform0051878	F-AATTTCAGCGCATGTGTGACAATT	134
				R-CCACCACCATGTCTTGTCTAGC	
		*HmCHS2*		F-AGGGCGCACGTGTTCTTGTT	125
			Isoform0001712	R-GATCACCGCTGATGCACCGT	
			Isoform0002955		
Chalcone isomerase	*CHI*	*HmCHI*	Isoform0062450	F-ATTGTTCCTCGGTGGCGCAG	124
			Isoform0064242	R-GCCCTTCCACTTAACGGCGA	
Flavanone 3-hydroxylase	*F3H*	*HmF3H1*	Isoform0002931	F-AGGGATGGTGGGAACACTTGG	89
				R-TTGCTCAGATAATGACCATGGTCG	
			Isoform0053472		
		*HmF3H2*	Isoform0056122	F-CCTCTTCTTTCAAGGAGGTGGTG	211
				R-CTCTGGTTCAGGACATGGTGG	
		*HmF3H3*	Isoform0060184	F-AACCCTCCTTCTTACAGAGAAGCT	135
				R-TTCACCACCACCAGCATCC	
Flavonoid 3′-hydroxylase	*F3*′*H*	*HmF3*′*H1*	Isoform0047984	F-AAGCCTTATTGATGGACATGGTGG	178
				R-TACGGGAGTTTTTGGATGTGGGAC	
		*HmF3*′*H2*	Isoform0013683	F-GCAACTCCGATTCGGCTCCT	119
			Isoform0014970	R-ACTTTCCGGCGGCGGTTTTA	
			Isoform0048829		
			Isoform0034725		
			Isoform0042732		
Flavonoid 3′5′-hydroxylase	*F3*′*5*′*H*	*HmF3*′*5*′*H*	Isoform0001658	F-AGGGCAAGCCGGACTTTCTT	109
				R-CCGGCAGTGAACAAATTCAAGAGTA	
Dihydroflavonol-4-reductase	*DFR*	*HmDFR1*	Isoform0047641	F-TCTCTGGAGCTCGCTTACGG	151
			Isoform0055957	R-TCAATCCTCCAACATTGACCGAG	
			Isoform0057874		
		*HmDFR2*		F-GCTGCCAAAGGCTGAAAAGAACT	146
			Isoform0054800	R-ACTTCATTCTCAGGGTCCTTGGA	
Anthocyanidin synthase	*ANS*	*HmANS1*		F-AGCCATCCACGGGAGTGCA	104
			Isoform0064501	R-CAAGCACCAATCTCTTCTCCTCCA	
		*HmANS2*	Isoform0002842	F-TGGTGATCAAATACAGGTTCTAAGC	98
			Isoform0004318	R-AGAAGGCAAGCGATACGCG	
			Isoform0048013		
		*HmANS3*	Isoform0054522	F-CCTCTCGGTGCTATCCCTCG	134
				R-CCTAGAGCGAGCTCCGGTTG	

**Table 2:** Correlation coefficients between the expression levels of genes and the contents of anthocyanins.

	*HmCHS1*	*HmCHI*	*HmF3H1*	*HmF3*′*H1*	*HmF3*′*5*′*H*	*HmDFR2*	*HmANS3*
Content of anthocyanins	0.91 ^1^	0.97 ^1^	0.97 ^1^	0.90 ^1^	0.91 ^1^	1.00 ^1^	0.96 ^1^

^1^ Indicates a significant correlation at the 0.01 level.

## Data Availability

The data that support the findings of this study have been deposited into the CNGB Sequence Archive (CNSA) of the China National GeneBank DataBase (CNGBdb) (https://db.cngb.org/cnsa/, accessed on 10 May 2022) with accession number CNP0003032 and NCBI Sequence Read Archive (SRA) of National Center for Biotechnology Information (https://submit.ncbi.nlm.nih.gov/subs/sra/, accessed on 10 May 2020) with accession number PRJNA849710. The mRNA sequence data have been submitted to the NCBI GenBank database under the accession number ON375338–ON375351.
